# Rasch analysis of the Patient Rated Elbow Evaluation questionnaire

**DOI:** 10.1186/s12955-015-0275-8

**Published:** 2015-06-20

**Authors:** Joshua I. Vincent, Joy C. MacDermid, Graham J. W. King, Ruby Grewal

**Affiliations:** Roth│McFarlane Hand and Upper Limb Centre, St. Joseph’s Healthcare London, Room DB 222, 268 Grosvenor Road, London, ON N6A 4 L6 Canada; School of Rehabilitation Science, McMaster University, 1280 Main Street West, Hamilton, ON L8S4L8 Canada; McFarlane Hand and Upper Limb Centre, St. Joseph’s Healthcare London, Room DB 222, 268 Grosvenor Road, London, ON N6A 4 L6 Canada; University of Western Ontario, Department of Surgery, London, ON Canada; Roth│McFarlane Hand and Upper Limb Center, St. Joseph’s Healthcare London, Room D0 213, 268 Grosvenor Road, London, ON N6A 4 L6 Canada; Department of Surgery, University of Western Ontario, London, ON Canada; Roth│McFarlane Hand and Upper Limb Center, St. Joseph’s Healthcare London, Room D0 209, 268 Grosvenor Road, London, ON N6A 4 L6 Canada

**Keywords:** Patient rated elbow evaluation, Rasch analysis, Elbow disorders, DIF, PSI, Chi-square, Fit residual

## Abstract

**Background:**

The Patient Rated Elbow Evaluation (PREE) was developed as an elbow joint specific measure of pain and disability and validated with classical psychometric methods. More recently, Rasch analysis has contributed new methods for analyzing the clinical measurement properties of self-report outcome measures. The objective of the study was to determine aspects of validity of the PREE using the Rasch model to assess the overall fit of the PREE data, the response scaling, individual item fit, differential item functioning (DIF), local dependency, unidimensionality and person separation index (PSI).

**Methods:**

A convenience sample of 236 patients (Age range 21–79 years; M: F- 97:139) with elbow disorders were recruited from the Roth│McFarlane Hand and Upper Limb Centre, London, Ontario, Canada. The baseline scores of the PREE were used. Rasch analysis was conducted using RUMM 2030 software on the 3 sub scales of the PREE separately.

**Results:**

The 3 sub scales showed misfit initially with disordered thresholds on17 out of 20 items), uniform DIF was observed for two items (“Carrying a 10lbs object” from specific activities subscale for age group; and “household work” from the usual activities subscale for gender); multidimensionality and local dependency. The Pain subscale satisfied Rasch expectations when item 2 “Pain – At rest” was split for age group, while the usual activities subscale readily stood up to Rasch requirements when the item 2 “household work” was split for gender. The specific activities subscale demonstrated fit to the Rasch model when sub test analysis accounted for local dependency. All three subscales of the PREE were well targeted and had high reliability (PSI >0.80).

**Conclusion:**

The three subscales of the PREE appear to be robust when tested against the Rasch model when subject to a few alterations. The value of changing the 0–10 format is questionable given its widespread use; further Rasch-based analysis of whether these findings are stable in other samples is warranted.

## Introduction

Quantifying pain and disability using patient-reported outcome measures (PROM) is an integral part in the evaluation of patients with any health condition. PROMs can be used to assess patient status, help set treatment goals and expectations; and more commonly to assess change following treatment interventions [1]. PROMs are used to assess outcomes in routine clinical practice, clinical research, and treatment trials because they provide a patient centered perspective which may differ from that provided by clinician based outcome measures (CBO) [2–5]. Currently, there are three different approaches to assessment of clinical measurement properties of rating scales 1) Traditional psychometric methods [6], 2) Rasch analysis [7] and 3) Item response theory (IRT) [8]. Rasch analysis is often considered as a one parameter model of IRT. It has been suggested that Rasch analysis has a greater potential to identify the strengths and weaknesses of rating scales than traditional psychometric methods [9]. The most important advantage of using a Rasch analysis is the capability of the analysis to convert ordinal level measurements into interval level measurements.

A majority of currently available PRO were developed prior to widespread use of Rasch and exist as an ordinal scale [10]. Issues have been raised with respect to the ability of these ordinal scales to provide a true quantitative scale that represents patient status along a continuum [10–12]. Forrest and Anderson [10] reported that when several items are measured on ordinal scales it is far from certain that the sum of scores has even ordinal properties. Merbitz et al. [12] suggested that ordinal scales of measurement do not support the mathematical operations needed to calculate means and standard deviations. One of the most important assumptions of parametric analysis is that the variables must have been measured in the interval scale, so that it is possible to interpret the results [13]. The Rasch model provides a potential solution by providing a means to transform non-linear ordinal score to become a (more) linear interval score, thus making the interpretation of the results possible and meaningful. However, it should be kept in mind that the raw scores remain ordinal even after Rasch analysis.

The Patient Rated Elbow Evaluation form (PREE) [14, 15] is a 20 item self-report measure, consisting of two sections, pain and function and the function section has two sub sections- ‘specific activities’ and ‘usual activities’. Responses are rated on a numeric rating scale. The pain section has five items of which four of them rate pain from ‘no pain’ (0) to ‘worst ever’ (10). The fifth item rates how often the patient has pain with responses ranging from ‘never’ (0) to ‘always’ (10). The responses on the function scale are anchored at ‘no difficulty’ (0) and ‘unable to do’ (10). The function section has 15 items regarding personal care, household work, occupation and recreational activities out of which 11 items fall under the specific activities sub-section and 4 items are under the usual activities sub-section. All the scores are computed to obtain a global score out of 100. Higher PREE total scores reflect greater pain and disability. The scaling of individual items was selected because 0–10 is easily comprehensible by patients and provides a range of scores [16]. The subscale structure was designed to reflect core concepts endorsed by patients and experts; and to be feasible in practice by emphasizing scoring simplicity as valued by users.

There are quite a few studies that have used traditional methods to evaluate the clinical measurement properties of the PREE. They have found the PREE to be valid with moderate to high correlations [14] The PREE has been found to have a very high level of internal consistency with Cronbach’s alpha values above 0.90 [17]. The PREE has shown high sensitivity to change [17, 18]. It has also demonstrated acceptable factor structure [17].

Rasch analysis is a relatively recent addition to the family of analyses used to test the psychometric properties of rating scales. Rasch analysis is the formal testing of how well items and questionnaires follow axioms of clinical measurement that are linked to a mathematical measurement model called the Rasch model [17]. During Rasch analysis, responses from a set of individual questions from a questionnaire can be tested against response patterns predicted by the model. The pattern expected by the model is a probabilistic form of Guttman structure which is a deterministic model that has a strict hierarchical ordering of items [19]. The PREE has not previously been subjected to Rasch analysis, meaning that interval level scaling has not been verified. Further, the potential for bias in different types of respondents has not been evaluated. Most studies using the PREE must assume interval level scaling or that parametric statistics are so robust that this will not affect results, since most rely on parametric statistics to make their conclusions, lack of interval level scaling or differential item functioning may lead to incorrect estimation of effects or false study conclusions. Since the PREE has demonstrated acceptable levels of clinical measurement properties using traditional methods and is commonly used with measurement of pain and disability arising from elbow disorders, it is important to evaluate its clinical measurement properties using modern psychometric methods.

Hence the purpose of the study is to conduct a Rasch analysis of the PREE to assess the overall fit to the Rasch model, the response scale used, individual item fit, differential item functioning (DIF), local dependency, unidimensionality and person separation.

## Methods

### Research design

Cross sectional study using Rasch analysis

### Participants

PREE scores were extracted from the charts of a cohort of 236 patients (Age range 21–79 years) who had completed outcome evaluations during surgical management of a variety of elbow conditions at the Roth│McFarlane Hand and Upper Limb Centre at St Joseph’s Healthcare in London, Ontario. Subjects were included in the study if they underwent a surgical intervention for elbow pathology, were aged 20 and above and had completed the PREE. Subjects with cognitive impairment and communication difficulties due to neurologic or psychiatric disorders were excluded from the study. The age distribution was as: 0 to 35 years (*n* = 51); 36 to 50 years (*n* = 100); 51 to 65 years (*n* = 65); 65 years and above (*n* = 20). There were 115 men and 121 women. The cohort included patients who have undergone biceps tendon repair, total elbow replacement arthroplasty, radial head fixation and radial head arthroplasty.

### Procedures

We selected the initial post-operative data point to conduct a cross-sectional analysis since this time point is commonly used in assessment; and we anticipate that there would be substantial variability in patient responses.

### Rasch analysis

Rasch analysis was performed using the RUMM 2030 software [20]. The 3 subscales of the PREE were analyzed separately for sources of misfit to the model using the analysis listed below. Since multiple testing was done, Bonferroni corrections were applied throughout the analyses as an adjustment. The steps laid out by Tennant and colleagues were followed [21].

#### Likelihood ratio test

There are 2 types of Rasch models that can be used with a polytomous dataset. They are the rating scale model [22] and the partial credit model [23]. The rating scale model constrains all thresholds of responses to be equally spaced across all of the items [24]. The partial credit model places no constraints on the threshold parameters.[25] To determine which model to use we first performed a formal test called the Likelihood-Ratio Test.[24] If the result of this test is not significant then the rating scale model would be used and if the result is significant then the partial credit model will be used 26]. We used a partial credit model based on a significant likelihood ratio test.

#### Inspection of class interval structure

The number of class intervals and the distribution of persons were inspected by looking for intervals to be approximately equally distributed [22].

#### Examination of the thresholds

Category probability curves were used to examine responses to an item [22]. Examination of the category probability curves can reveal disordered thresholds, meaning inconsistent use of response items by the respondents. This is a common source of item misfit. Disordered thresholds occur when respondents have difficulty consistently discriminating between response options [26–29]. Potential solutions for correcting disordered thresholds include collapsing of the categories to improve the overall fit to the model [30].

#### Fit statistics

The following important fit statistics are inspected when assessing the fit of the data to the Rasch model.

##### Item/person fit residuals

This tests the degree to which the Guttman pattern is achieved.[31] The individual item and person–fit statistics are expressed as residuals. To say that the item and person fit the model we expect the residuals to range between + 2.5 and −2.5 [32].

##### Item-trait interaction

This is tested to assess the property of invariance across the trait and is reported as a chi-square [33]. If the chi-square value is significant, this supports the presence of variance across the trait for hierarchical ordering of the items, compromising the required property of invariance [34].

#### Reliability indices

The Person-Separation-Index (PSI) [33, 35] indicates the ability of the construct to discriminate amongst the respondents. The value of 0.7 is considered by convention to be the minimum acceptable level of PSI. The PSI determines the number of groups of patients between whom we can statistically differentiate. A value of 0.8 is representing the ability to statistically differentiate at least 3 groups. A value of 0.9 would indicate the ability to discriminate between 4 or more groups [36–38]. PSI is an indicator of how much we can rely on the fit characteristics [38, 39]. Lower PSI indicates less reliability [38].

#### Differential item functioning (DIF)

DIF is another potential source of item bias resulting in misfit of the data to the model. Despite different groups (e.g., males/females) being at equal levels of the underlying trait, they may respond to an item differently, indicating a bias between the groups. DIF can be detected graphically (Item characteristic curves) and statistically (ANOVA). Uniform DIF is indicated by a significant main effect for the person factor (gender in this case), while the presence of non-uniform DIF is indicated by a significant interaction effect (gender x class interval) [37]. Sex-gender bias can occur because females and males interpret items differently, have different roles/expectations with respect to the item content or because items represent a different physiologic load based on sex-based differences. Similar issues can cause DIF by age. There are 2 types of DIF- a) Uniform DIF, where the group shows a consistent systematic difference in their responses to an item, across the whole range of the attribute being measured; b) When there is non-uniformity in the differences between the groups (e.g., it varies across levels of the attribute) then this is referred to as non-uniform DIF [31]. With Uniform DIF the problem can be remedied by splitting the file by group and separately calibrating the item for each group. Non-uniform DIF is more problematic because there is no mathematical adjustment; and typically it would require removing the item from the scale [31]. We assessed DIF for gender and age groups.

#### Local dependency

A violation of local independence occurs when examinee item responses depend not just on their trait level, but on their responses to other test items [22]. Principal component analysis (PCA) [40] of the residuals was done as a test for local independence. An inter-item residual correlation > 0.3 above the average residual correlation was used as a cut-off to indicate local dependency [41, 42]. The residuals were inspected visually. And the lack of any meaningful pattern in was taken as an indicator of local independence and consequently unidimensionality of the scale [40].

#### Unidimensionality

This was formally tested by the method proposed by Smith where we allow the factor loadings on the first residual to determine subsets of items and then testing, by a paired *t* test, to see if the person estimates derived from these subsets are significantly different [43]. We expect the percentage of tests that are significant (P < 0.05) should be less than 5 %, for the questionnaire to be unidimensional.

#### Targeting

Every questionnaire should be well-targeted towards the patient population in question. In other words the thresholds should cover a range a difficulties and the targeted population should fall within a similar range of abilities. This was analyzed by plotting the person-item location threshold distribution graph with distributions of persons on the top half of the graph and item thresholds at the bottom half of the graph. The average item difficulty is always calibrated at zero logits, therefore the average person location of zero logits would indicate a fairly good targeting [43].

## Results

There was no missing data and all 236 cases were determined to be valid by the RUMM 2030 software. The 3 sub scales were analysed separately. The class intervals were checked throughout the analysis for consistency and the cases were nearly equally distributed between the groups (See Table 1, initial analysis).

**Table 1:** Summary fit statistics for individual subscales of the PREE

Analysis	Item fit residual		Person Fit residual		Item-trait interaction		Unidimensionality	PSI
	Mean	SD	Mean	SD	Chi square *(df)*	*P*	Per C < 5 % (95 % C.I)	
Pain subscale								
Initial	−0.01	1.02	−0.34	0.88	13.77 (15)	0.001*	5 % (2 % - 7 %)	0.87
Final	−0.09	0.94	−0.34	0.87	17.87 (18)	0.47	- (since items were split for DIF)	0.90
Specific activities subscale								
Initial	0.08	1.27	−0.32	1.21	55.96 (33)	0.01*	1 % (7 % –13 %)*	0.83
Final	0.05	1.82	−0.39	1.08	10.87 (15)	0.76	3 % (1 % – 6 %)	0.91
Usual activities subscale								
Initial	−0.72	0.56	−0.41	0.94	13.39 (12)	0.34	2 % (1 % - 5 %)	0.82
Final	−0.55	1.02	−0.44	1.02	6.01 (10)	0.82	-(since items were split for DIF)	0.86

*Source of misfit to the Rasch model; SD = Standard deviation; df = Degrees of freedom; per C < 5 % = proportion of t tests that were significant at level of significance of 0.05; 95 % CI = 95 % confidence interval; PSI = Person separation index; PREE – Patient Rated Elbow Evaluation

• For the data to satisfy Rasch model requirements

• Mean is expected to be approx. around zero (Can range between + 2.5 to −2.5)

• S.D. should be approx. 1

• Chi square value is expected to be small and statistically non-significant

• For a measure to display evidence of unidimensionality, less than 5 % of the t-tests should be significant at *p* = 0.05. If more than 5 % of the tests are significant, then the lower bound of the 95 % confidence interval should be less than 5 % to offer some support of unidimensionality

• PSI (Person separation index) PSI should be greater than 0.7 to obtain good power for the tests of fit

### Handling of data to fit the Rasch model

#### Pain subscale

Analysis of the 5 items of the pain sub scale revealed slight deviation from the Rasch model requirements as indicated by a high and significant item trait interaction (*p* < 0.001). (See Table 1) Items 3, 4 and 5 exhibited disordered thresholds. Individual item fit was excellent indicating acceptable levels of discrimination. (See Table 2) Uniform DIF for age group was observed for item 2, “*Pain -* At rest”. (See Table 3) Unidimensionality was acceptable (See Table 1; initial analysis). The Reliability Index was high with a PSI of 0.87. No meaningful pattern of local dependency was observed.

**Table 2:** Initial fit statistics for individual items of the PREE

Item	Location	SE	Fit statistics		
			Fit residual	Chi Square	Chi square probability
Pain sub scale					
Pain - When it is at its worst	−0.32	0.05	0.88	1.62	0.66
Pain - At rest	1.32	0.04	−1.10	2.05	0.56
Pain - When lifting a heavy object	−0.67	0.06	1.05	3.85	0.28
Pain - When doing a task with repeated elbow movement	−0.36	0.05	−1.28	4.35	0.23
How often do you have pain?	0.03	0.06	0.06	1.87	0.60
Specific activities sub scale					
Comb my hair	0.50	0.08	−1.42	7.70	0.05
Eat with a fork or spoon	0.55	0.08	−1.26	4.15	0.25
Pull a heavy object	−1.25	0.09	−0.91	1.07	0.78
Use my arm to rise from a chair	−0.35	0.07	−0.12	3.08	0.38
Carry a 10 lb object with my arm at my side	−0.82	0.07	2.29	13.61	0.03
Throw a small object, such as a tennis ball	−0.60	0.08	0.96	0.24	0.97
Use a telephone	0.63	0.07	1.03	5.69	0.13
Do up buttons on the front of my shirt	0.42	0.07	0.91	2.64	0.45
Wash my opposite armpit	0.44	0.08	−1.04	6.41	0.09
Tie my shoe	0.37	0.10	1.27	6.19	0.10
Turn the doorknob and open a door	0.10	0.08	0.56	1.43	0.70
Usual activities sub scale					
Personal activities (dressing, washing)	0.86	0.06	−0.97	1.91	0.39
Household work (cleaning, maintenance)	−0.04	0.05	−2.17	3.95	0.14
Work (your job or everyday work)	−0.35	0.07	0.25	2.01	0.37
Recreational activities	−0.47	0.07	0.65	0.12	0.94

SE-standard error; PREE – Patient Rated elbow Evaluation; ^a^ Was not significant after Bonferroni correction applied

**Table 3:** DIF summary (Age Group) for the individual items of the PREE

Item	Uniform DIF for Age				Non-Uniform DIF for Age			
	MS	F	DF	*P*	MS	F	DF	*P*
Pain sub scale								
Pain - When it is at its worst	1.55	1.77	3	0.15	0.92	1.05	9	0.40
Pain - at rest^a^	2.98	4.82	3	0.00	0.79	1.28	9	0.25
Pain - When lifting a heavy object	1.29	1.36	3	0.26	0.40	0.42	9	0.93
Pain - When doing a task with repeated elbow movement	1.31	2.02	3	0.11	0.44	0.68	9	0.73
How often do you have pain?	0.89	1.11	3	0.35	0.63	0.78	9	0.64
Specific activities sub scale								
Comb my hair	0.38	0.58	3	0.63	1.38	2.08	9	0.03
Eat with a fork or spoon	0.62	0.90	3	0.50	0.84	1.20	9	0.30
Pull a heavy object	1.23	1.58	3	0.20	0.50	0.64	9	0.76
Use my arm to rise from a chair	1.25	1.41	3	0.24	0.45	0.51	9	0.86
Carry a 10 lb object with my arm at my side	3.96	3.32	3	0.02	2.20	1.84	9	0.06
Throw a small object, such as a tennis ball	1.74	1.69	3	0.17	1.14	1.11	9	0.35
Use a telephone	0.99	0.94	3	0.42	0.94	0.89	9	0.54
Do up buttons on the front of my shirt	2.05	1.99	3	0.12	0.65	0.63	9	0.77
Wash my opposite armpit	1.94	2.54	3	0.06	0.21	0.27	9	0.98
Tie my shoe	0.29	0.27	3	0.84	0.57	0.54	9	0.85
Turn the doorknob and open a door	1.39	1.46	3	0.22	1.05	1.10	9	0.36
Usual activities sub scale								
Personal activities (dressing, washing)	0.07	0.10	3	0.96	0.12	0.19	6	0.98
Household work (cleaning, maintenance)	1.38	2.83	3	0.04	0.33	0.67	6	0.67
Work (your job or everyday work)	0.95	1.28	3	0.28	1.33	1.81	6	0.10
Recreational activities	2.41	2.99	3	0.03	0.47	0.58	6	0.74

^a^Items exhibiting Uniform DIF. An item was considered to exhibit DIF if *P* values are significant after applying Bonferroni correction factor; PREE – Patient Rated Elbow Evaluation

**Fig. 1:**
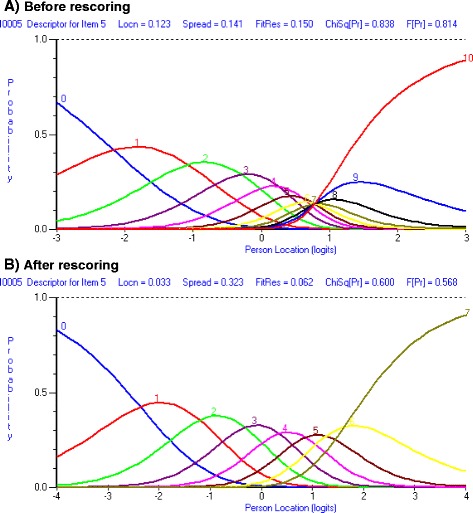
Showing disordered threshold for item 5 “How often do you have pain?” of the pain subscale **a** Before rescoring, **b** After rescoring

**Fig. 2:**
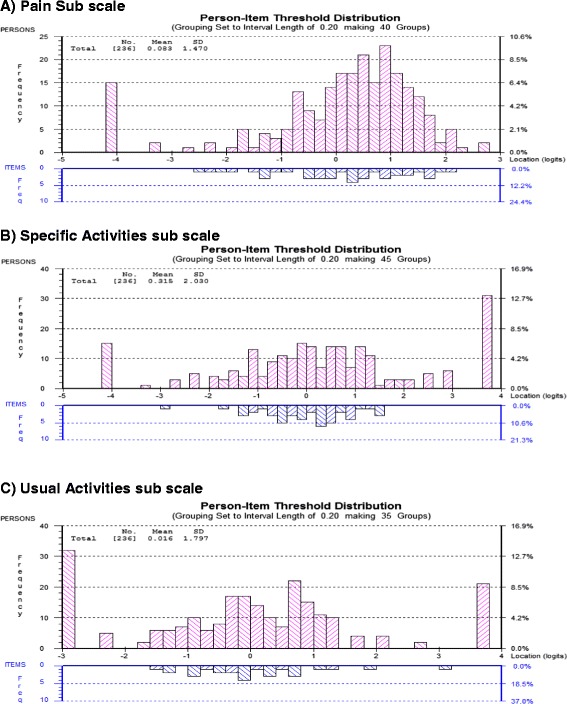
Person-item threshold distributions for the individual subscales of the Patient Rated Elbow Evaluation questionnaire showing targeting (Final analysis) **a** Pain sub scale **b** Specific activities sub scale, **c** Usual avctivities sub scale

To improve the overall fit to the Rasch model items 3, 4, and 5 were rescored to a 0–7 scale. (See Table 4; Fig 1) Then item 2 was split for age group; this resulted in excellent item fit and non-significant item trait interaction. Uniform DIF (Age group) for item 2 was not evident Table 5. Unidimensionality was observed and no local dependency was present Table 6. The reliability improved to be 0.90. (See Table 1; final analysis) In spite of some floor and ceiling effects observed, targeting was also good as indicated by the person item threshold map. (See Fig. 2a) This implies that this sub scale has a good coverage for elbow disorders related pain. Hence, this was accepted as the final model.

**Table 4:** Table showing the structure of scores for individual items of the PREE

Item	0	1	2	3	4	5	6	7	8	9	10
Pain sub scale											
Pain - When it is at its worst	0	1	2	3	4	5	6	7	8	9	10
Pain - At rest	0	1	2	3	4	5	6	7	8	9	10
Pain - When lifting a heavy object^a^	0	1	1	2	3	4	5	5	6	6	7
Pain - When doing a task with repeated elbow movement^a^	0	1	2	3	4	4	5	5	6	6	7
How often do you have pain?^a^	0	1	2	3	4	4	5	5	6	6	7
Specific activities sub scale											
Comb my hair^a^	0	1	1	1	2	2	2	3	3	3	4
Eat with a fork or spoon^a^	0	1	1	1	2	2	3	3	3	3	4
Pull a heavy object^a^	0	1	1	1	2	2	2	3	3	3	4
Use my arm to rise from a chair^a^	0	1	1	2	2	3	3	4	4	4	5
Carry a 10 lb object with my arm at my side^a^	0	1	2	2	3	3	4	4	4	4	5
Throw a small object, such as a tennis ball^a^	0	1	1	2	2	2	3	3	3	3	4
Use a telephone^a^	0	1	1	2	2	3	3	3	4	4	5
Do up buttons on the front of my shirt^a^	0	1	1	2	2	3	3	3	4	4	5
Wash my opposite armpit^a^	0	1	1	1	2	2	2	3	3	3	4
Tie my shoe^a^	0	1	1	1	1	2	2	2	2	2	3
Turn the doorknob and open a door^a^	0	1	1	2	2	2	2	3	3	3	4
Usual activities sub scale											
Personal activities (dressing, washing)^a^	0	1	1	2	2	3	3	4	5	6	7
Household work (cleaning, maintenance)	0	1	2	3	4	5	6	7	8	9	10
Work (your job or everyday work)^a^	0	1	1	2	2	3	3	4	4	4	5
Recreational activities^a^	0	1	1	2	2	3	3	3	4	4	5

^a^Rescored items; PREE – Patient Rated Elbow Evaluation

**Table 5:** DIF summary (gender) for the individual items of the PREE

Item	Uniform DIF for Gender				Non-Uniform DIF for Gender			
	MS	F	DF	*P*	MS	F	DF	*P*
Pain sub scale								
Pain - When it is at its worst	3.76	4.31	1	0.04	0.88	1.01	3	0.39
Pain - At rest	0.83	1.28	1	0.26	1.00	1.53	3	0.21
Pain - When lifting a heavy object	0.00	0.00	1	0.96	0.10	0.10	3	0.96
Pain - When doing a task with repeated elbow movement	1.58	2.43	1	0.12	0.07	0.11	3	0.95
How often do you have pain?	0.40	0.50	1	0.48	0.50	0.62	3	0.61
Specific activities sub scale								
Comb my hair	3.25	4.76	1	0.03	0.29	0.42	3	0.74
Eat with a fork or spoon	0.10	0.14	1	0.71	1.37	1.97	3	0.12
Pull a heavy object	0.00	0.00	1	0.97	0.15	0.19	3	0.90
Use my arm to rise from a chair	1.52	1.77	1	0.19	1.20	1.39	3	0.25
Carry a 10 lb object with my arm at my side	0.11	0.08	1	0.78	0.79	0.60	3	0.61
Throw a small object, such as a tennis ball	3.34	3.35	1	0.07	3.08	3.08	3	0.03
Use a telephone	0.90	0.84	1	0.36	0.53	0.49	3	0.69
Do up buttons on the front of my shirt	0.04	0.04	1	0.84	2.73	2.72	3	0.05
Wash my opposite armpit	0.51	0.67	1	0.41	0.37	0.49	3	0.69
Tie my shoe	0.49	0.47	1	0.49	0.20	0.19	3	0.91
Turn the doorknob and open a door	0.00	0.00	1	0.99	2.11	2.21	3	0.09
Usual activities sub scale								
Personal activities (dressing, washing)	0.65	1.03	1	0.31	−0.16	−0.25	2	0.99
Household work (cleaning, maintenance)^a^	6.68	14.28	1	0.00	−0.06	−0.14	2	0.99
Work (your job or everyday work)	3.24	4.35	1	0.04	0.72	0.97	2	0.38
Recreational activities	3.74	4.61	1	0.03	0.27	0.33	2	0.72

^a^Items exhibiting Uniform DIF. An item was considered to exhibit DIF if *P* values are significant after applying Bonferroni correction factor; PREE – Patient Rated Elbow Evaluation

**Table 6:** Principal component analysis (PCA) showing first component loadings for individual items of the PREE

Item	Principal component 1
Pain sub scale	
Pain - When it is at its worst^a^	0.01
Pain - At rest	−0.69
Pain - When lifting a heavy object^a^	0.74
Pain - When doing a task with repeated elbow movement^a^	0.55
How often do you have pain?^a^	−0.43
Specific activities sub scale	
Comb my hair^a^	0.12
Eat with a fork or spoon^a^	0.09
Pull a heavy object	−0.69
Use my arm to rise from a chair	−0.42
Carry a 10 lb object with my arm at my side	−0.63
Throw a small object, such as a tennis ball	−0.33
Use a telephone^a^	0.35
Do up buttons on the front of my shirt^a^	0.74
Wash my opposite armpit^a^	0.59
Tie my shoe^a^	0.45
Turn the doorknob and open a door	−0.13
Usual activities sub scale	
Personal activities (dressing, washing)	−0.61
Household work (cleaning, maintenance)	−0.65
Work (your job or everyday work)^a^	0.68
Recreational activities^a^	0.72

^a^Positively loaded items; PREE – Patient Rated Elbow Evaluation

#### Specific activities subscale

Rasch analysis revealed that the 11 item specific activities subscale has marked deviations from the Rasch model expectations. This was evident from, the disordered thresholds (11 out of the 11 items); the property of invariance was compromised because of large and significant chi square value that was observed. There was a breach of unidimensionality as well. (See Table 1; initial analysis) Local dependency was observed between the following items, Item 1 “*Comb my hair*”; Item 2 “*Eat with a fork or spoon*”; Item 3 “Pull a heavy object”; Item 4 “Use my arm to rise from a chair”; Item 5 “*Carry a 10 lb object with my arm at my side*”; Item 7 “Use a telephone”; Item 8 “Do up buttons on the front of my shirt”; Item 9 “*Wash my opposite armpit*”; Item 10 “*Tie my shoe*”. The pairs that exhibited local dependency are as follows: 1 & 2, 3 & 4, 3 & 5, 7 & 8, 8 & 9, 8 &10 and 9 & 10. DIF analysis revealed that none of the items exhibited DIF for age group or gender (See Table 3 and 5). Individual item fit was excellent indicating acceptable levels of discrimination. (See Table 2) and the reliability of the scale was high (PSI = 0.83) (See Table 1; initial analysis).

To improve the fit of the specific activities subscale to Rasch model various actions were taken. Initially the 11 items with disordered thresholds were rescored. Thresholds were more disordered in the middle of the 0–10 scale. So categories were collapsed to a 5 point or a 6 point scale depending on the item. (See Table 4) To deal with local dependency, subtest analyses was done to see if they can be accounted for at the sub test level. Testlets were created by combining items 1 and 2; 3, 4 and 5; and 8, 9 and 10. Items 1 & 2 were combined as they are both items of instrumental activities of daily living (Self-care) above the level of shoulder; items 3, 4 & 5 were combined as they are activities that produce high levels of forces around the elbow and lastly we combined 8, 9 and 10 as they are all self-care activities. When the subtest analysis was completed local dependency was accounted for and the chi square residual became non-significant indicating acceptable fit of the data to the Rasch model. Unidimensionality was observed. The reliability improved to be 0.91. (See Table 1; final analysis) Targeting was acceptable with enough coverage; also some floor and ceiling effects were observed (See Fig. 2b).

#### Usual activities subscale

The usual activities subscale initially demonstrated misfit to the Rasch model with disordered thresholds for three of the four items (items1, 3 and 4). There was no DIF for age group. Uniform DIF for gender was observed for item 2 “*Household work (cleaning, maintenance)”*. (See Table 5) There was no breach of the properties of invariance, local independence and unidimensionality. Reliability was acceptable (PSI = 0.82). (See Table 1; initial analysis)

To improve the fit of the scale to the Rasch model the items with disordered thresholds were rescored to reorder them. (See Table 4). To deal with DIF for gender, item 2 was split for gender. The final analysis rendered the data to fit the Rasch model, increasing reliability of the sub scale (PSI = 0.86) and bringing down the chi square value. (See Table 1; final analysis) The scale was well targeted as demonstrated by the person-item threshold map; however, some floor and ceiling effects were evident (See Fig. 2c).

## Discussion

The results of this Rasch analysis support the claims made by classical test methods on the psychometric properties of the PREE that is has acceptable measurement properties [22], but also suggests that there are potential areas of improvement in scoring for the PREE to derive an unbiased patient reported estimate of pain and disability in elbow disorders. The stability of these findings at different time points and in different samples is unknown and so decisions about changes to the PREE may be premature, but the findings suggest considerations of optimization and application of the PREE.

Ideally measures would be developed using Rasch analysis, but many commonly used measures, including the PREE, pre-date the common use of Rasch- and were developed and validated using a traditional clinimetric approach. Therefore, some lack of fit to Rasch is often found when investigating a clinimetrically valid PRO, including other measures in the Patient-rated family for the wrist [44]. The PREE exhibited acceptable level of fit to the Rasch model requirements with less complicated data handling. By assessing the fit of the PREE data to the Rasch model, and following a sequential Rasch approach to assess potential sources of misfit we have identified areas that need to be improved to achieve a linear interval score. These interval scores can accurately reflect change in patient disability status; whereas an ordinal scale cannot [44].

The PREE had 17 items (3 items from the pain sub scale; 11 from the specific activities; 3 from usual activities) with disordered thresholds out of the 20 items. This draws our attention to the 0 to 10 numeric rating scale (an ordinal scale) used in this self-report measure. Similar findings have been observed in the Patient Rated Wrist and Hand Evaluation (PRWHE), [13] the wrist and hand counterpart of the PREE. While the 0–10 scale is commonly used and accepted by patients, 11 response options may exceed what patients can discriminate as distinct levels [44]. Another possibility is that the items are too difficult for the patients to calibrate. However, during development of this measure, patients preferred the 0–10 scale as they it found easier to respond to; and found the items easy to understand [23, 45]. Furthermore, the PREE was shown to be well-targeted with a person-item location slightly less than the average of zero logits, which discounts item difficulty as a problem (See Fig. 2). Rescoring of these items as indicated in Table 4 places additional burden on the clinician but may retain ease of administration and patient acceptance. With computer administration, background scoring algorithms can be implemented without changing the “face” of an instrument. Alternatively the scaling can be redesigned to be a 6 point (0–5) scale that is used in both electronic and print versions. A downside to this solution is that, it might be challenging to select the right descriptors that would be meaningful to patients for all items on the 3 subscales. The 0–10 scale is commonly used in clinical practice and is more sensitive and easily understood than VAS scales [14] can present the same discrimination challenges to patients. Finally, such a substantial change on a measured that has performed well in many other contexts on the basis of one study might be preliminary- particularly since changes to scoring were able to address most measurement concerns. Therefore, it seems that rather than changing the scale, a background Rasch scoring algorithm might be a preferable approach. However, what yet remains to be determined about Rasch-based alternate scoring for measures is the extent to which it makes a difference in the applications for measurement.

In all three sub scales none of the items demonstrated a misfit as indicated by fit residuals that were within acceptable limits. (See Table 2) This indicates that none of the items were over discriminating. We observed large and significant chi square initially for the specific activities sub scale indicating the presence of a latent trait violating the property of invariance. However this got adjusted when testlets were created in the sub test analysis.

To satisfy the assumptions of unidimensionality it is suggested that the three sub scales of the PREE be considered separately. Scoring pain and disability subscales separately are aligned with the developer’s original intention of having these subscales and establishing scale reliability [46]. It is in agreement with recommendations for the similar PRWE both based on Rasch analysis [44] and expert consensus [14]. However, many studies continue to report the total score of pain and disability measures, perhaps because having a single primary outcome measure is preferred for study design and interpretation. Where such a composite score is used, the user should be careful to analyse the deconstructed measure and insure that conclusions are not affected by pooling.

Unidimensionality was not an issue with the pain and usual activities subscale. However, the specific activities subscale exhibited multidimensionality. This confirms the observations made through an exploratory factor analysis where the specific activities subscale loaded onto more than 1 factor [47]. The cause for multidimensionality was local dependency observed between the items. This local dependency was accounted for when subtest analysis was performed. This indicates that there are some redundant items in the specific activities subscale that could potentially be removed. Since the measure is established and brief, the benefits of this would need consideration. There would be limited time savings to such a step and it might complicate the scoring.

In the pain sub scale of the PREE, the item “Pain: At rest” was the source of misfit. This item demonstrated a uniform DIF for age group. This is not surprising as previous basic science research findings indicate that pain tolerance is reduced as people age suggesting the possibility that older people might perceive their pain levels differently than younger ones as they did in, our sample [17]. Uniform DIF for gender was observed for the item “Household work (cleaning, maintenance)” (*p* = 0.001) under usual activities of the function subscale. There can be gender-based differences in “household work’ with men usually performing heavier household tasks while women tend to do lighter tasks [48–50] but a greater portion of the work [51]. This may explain why men and women answered this question differently. Gender was considered as a potential source of differential response when designing this scale (which pre-dated Rasch) [52] and thus the items specified both cleaning and maintenance to embrace different household roles. However, being inclusive cannot guarantee that the item will be perceived and calibrated the same way by both genders. We recommend that future studies evaluate the extent and source of gender differences in responding to the PREE items. Since we only examined differential item functioning based on gender and age group, there is a need to conduct examination for other potential sources including affected side. More clinical constructs can be added to the DIF analysis to see how the individual items behave with the different constructs. Since gender and age are commonly reported in clinical research studies, the distributions of these may need to be considered when interpreting the PROM reported in clinical studies in patients with elbow conditions that use the PREE or other measures where Rasch has not been used with insure interval level scaling.

With the increasing use of Rasch, new flaws are being detected in many PROMs that were developed using more traditional clinimetric approaches. This has potential to improve clinical measurement by improving or discarding tools that do not provide valid measurement. However, we suggest a cautious approach in suggesting changes to measures. Different Rasch analyses on the same scale across different studies have reported different findings and made different recommendations about what changes should be made to make the measure “better” [14]. We found that that changing the scale scoring to meet Rasch based interval level scaling can have an impact on study conclusions, [53–55] but few others have undertaken such evaluations when proposing that scores need to be changed. When the threshold for changing PROM is low, this can result in multiple variants of a PRO, with no clear choice of the best option. The potential benefits to change the scale must be weighed against the well documented knowledge translation challenges in implementation of PROM [56, 57] and need for consistency across comparisons. Hence, we suggest that where findings are consistent with previous psychometric findings and support the current PREE (with item rescoring) then this warrants continued use of the current PREE. Where we have found suboptimal measurement findings that are not consistent with that reported in other studies or across time-points we suggest caution and further study.

The strengths of the current study are its high PSI values and using a heterogeneous group of patients. The limitations of the current study are: not all elbow disorders were represented and that we looked at the DIF only for gender and age. Our sample size was moderate; however our power of fit was excellent. Another limitation is that we were not able to provide a transformation table showing the Rasch converted scores which would allow interval level measurement. Given our position that a sufficient preponderance of stable evidence is needed to warrant changing a well-established PRO that has substantial psychometric support in traditional analyses. Thus, the lack of tools to accomplish this is consistent with our view on the burden of evidence required to propose permanent changes. We were also not able to perform a longitudinal analysis using the Rasch software. We recommend future studies carry out a longitudinal analysis to assess the responsiveness of PREE. We also recommend future studies include a variety of elbow disorder patients; evaluate other potential sources of differential item functioning such as occupational demand, severity of injury, level of education, worker’s compensation claim and other social factors that might determine the DIF. Our findings questioned the measurement properties of the items of the specific activities subscale. It might be worthwhile exploring the stability of our findings before implementing substantial changes- particularly in light of the strong psychometric properties demonstrated in previous studies using classical test methods.

## Conclusion

All the three sub scales of the PREE appear to be robust when tested against the Rasch model amenable to few changes. Rasch analysis has highlighted areas needing further investigations and potential modification of the rating scale due to the misfit caused by disordered thresholds in our sample. Additional studies are needed to assess the consistency of item performance across contexts that will lead to an optimal format and scoring of the PREE based on a preponderance of findings.
